# Thoracic aortic wall shear stress atlases in patients with bicuspid aortic valves

**DOI:** 10.1186/1532-429X-16-S1-P161

**Published:** 2014-01-16

**Authors:** Pim van Ooij, Wouter V Potters, Aart J Nederveen, Jeremy D Collins, James C Carr, SC Malaisrie, Michael Markl, Alex J Barker

**Affiliations:** 1 Radiology, Northwestern University, Chicago, Illinois, USA; 2 Radiology, Academic Medical Center, Amsterdam, Netherlands; 3 Medicine-Cardiology, Northwestern University, Chicago, Illinois, USA; 4 Biomedical Engineering, Northwestern University, Chicago, Illinois, USA

## Background

Wall shear stress (WSS) may be associated with the onset and development of aortopathy in the presence of bicuspid aortic valve (BAV) [Michelena, JAMA (2011)]. The use of 'aortic atlases' of 3D WSS vectors allows for systematic analysis of WSS differences between large patient cohorts. In this study, a comparison is performed between WSS atlases of BAV patients, BAV patients with aortic dilation, patients with BAV stenosis, and healthy controls with tricuspid valves. The aim is to test the hypothesis that WSS atlases can identify regions of significantly altered WSS that are associated with different expression of BAV and aortopathy.

## Methods

Prospectively ECG and respiratory gated 4D flow MRI was performed in 10 healthy controls, 10 BAV patients, 10 BAV patients with aortic dilation (as defined by aorta diameter > 4 cm), and 10 patients with BAV stenosis (table 1) on 1.5 and 3T systems (Siemens, Erlangen, Germany). Imaging parameters were: resolution = 1.7-3.6 × 1.7-2.4 × 2.2-3.2 mm3, temporal resolution = 37-43 ms, TE/TR/FA/VENC = 2.2-2.8 ms/4.6-5.4 ms/7-15°/150-300 m/s. The data were corrected for Maxwell terms, eddy currents and velocity aliasing. Segmentation of the aorta was performed in MIMICS (Materialise, Leuven, Belgium) using PC-MRA images. WSS along the entire segmented aorta surface was calculated as previously described [van Ooij, JMRI (2013)]. WSS atlases were created as follows: the segmentations of the aorta for each cohort were co-registered. By maximizing the degree of overlap, the geometry that showed the smallest deviation with the average aorta shape in the cohort was chosen. The WSS vectors of each subject were interpolated onto this geometry and averaged over all subjects, resulting in the cohort specific WSS atlas. Mean WSS was calculated in the ascending aorta (AAo), arch and descending aorta (DAo). For statistical analysis, a shared geometry was created from the 4 atlas geometries and each individual aortic WSS was interpolated to this shared geometry.

**Table 1:** Age, aortic diameter and mean WSS in the AAo, arch and DAo of the cohort-specific WSS atlases.

	Controls	BAV	BAV with Dilation	BAV Stenosis
Age+ (y)	50 ± 14	44 ± 10	53 ± 10	44 ± 10

SOV diameter* (cm)	3.0 ± 0.5	3.5 ± 0.4	4.0 ± 0.3	3.7 ± 0.4

MAA diameter* (cm)	2.9 ± 0.5	3.4 ± 0.4	4.4 ± 0.3	3.4 ± 0.4

Mean WSS AAo* (Pa)	0.51 ± 0.16	0.55 ± 0.24	0.71 ± 0.46	0.61 ± 0.37

Mean WSS arch* (Pa)	0.50 ± 0.14	0.63 ± 0.18	1.05 ± 0.27	0.73 ± 0.32

Mean WSS Dao* (Pa)	0.64 ± 0.10	0.64 ± 0.12	1.16 ± 0.27	0.67 ± 0.14

Comparison		BAV vs. Controls	BAV with dilation vs. Controls	BAV stenosis vs. Controls

Percentage significant difference AAo (%)**		28	41	37

Percentage significant difference arch (%)**		20	86	35

Percentage significant difference DAo (%)**		0	67	0

Median/mean angle AAo (°)*		23/32	34/46	26/43

Median/mean angle arch (°)*		9/13	14/19	15/19

Median/mean angle DAo (°)*		6/6	6/7	6/7

Percentage significant difference shows the percentage of the area of the AAo, arch and DAo, where the difference with the control atlas on the shared geometry was significant. The median/mean angle represents the median and mean of the angle difference of the WSS vector with the control atlas on the shared geometry.

SOV = Sinus of Valsalva, MAA = Mid-Ascending aorta, AAo = Ascending aorta, DAo = Descending aorta + Not significant across cohorts using a Kruskal-Wallis test (p = 0.3) *Significant across cohorts using a Kruskal-Wallis test (p < 0.05) ** Significance tested with a Wilcoxon rank sum test (p < 0.05)

## Results

**Figure 1:**
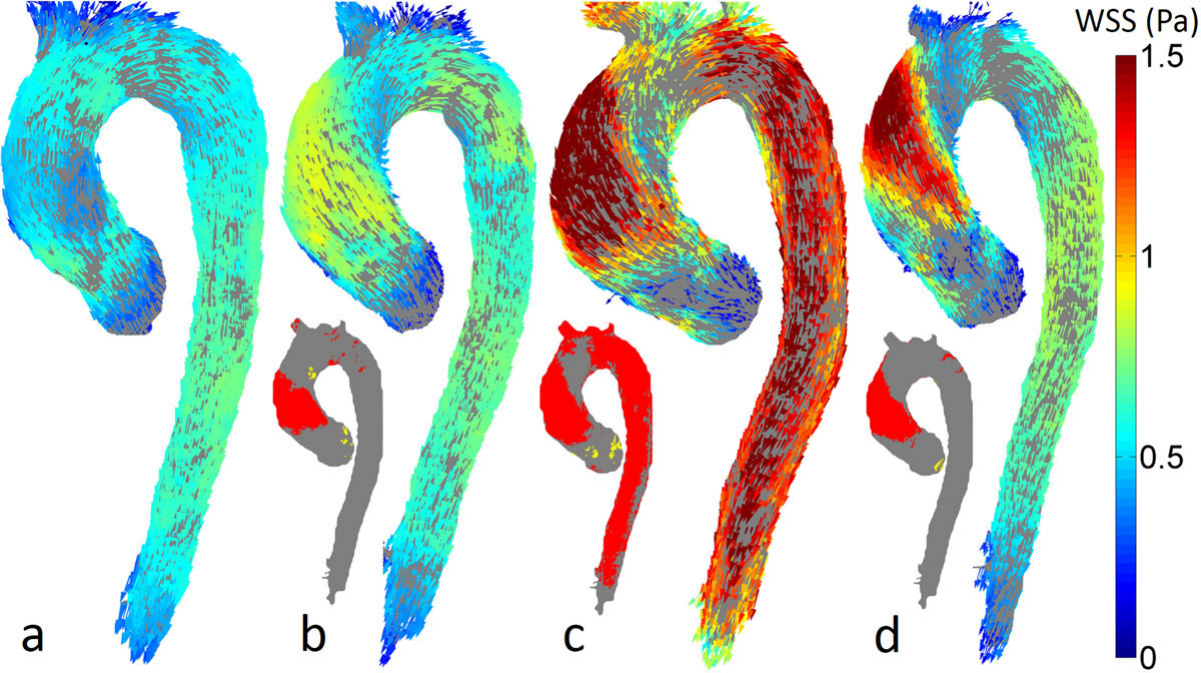
**Cohort-specific WSS atlases of (a) healthy controls with tricuspid valves, (b) BAV patients, (c) BAV patients with aortic dilation, (d) patients with BAV stenosis**. The insets show the p-value maps on the shared geometry of the BAV vs. controls (b), BAV with dilation vs. controls (c) and BAV stenosis vs. controls (d) comparison.

Figure 1 shows the cohort-specific WSS atlases. WSS was significantly higher for all BAV cohorts compared to the control atlas (Table 1). The BAV with dilation cohort showed the highest WSS which was significantly increased compared to the control atlas in a large part of the aorta (Table 1). Furthermore, WSS direction showed the highest deviation in the AAo (median: 46°) from the control atlas for the BAV with dilation cohort.

## Conclusions

WSS atlases demonstrate the ability to regionally detect that all BAV patients exhibit significantly elevated WSS compared to healthy volunteers with tricuspid valves.

## Funding

NIH NHLBI grant R01HL115828; American Heart Association Scientist Development Grant 13SDG14360004.

